# Genetic regulation of lateral root development

**DOI:** 10.1080/15592324.2022.2081397

**Published:** 2022-06-01

**Authors:** Ying Zhang, Yuru Ma, Dan Zhao, Ziyan Tang, Tengteng Zhang, Ke Zhang, Jingao Dong, Hao Zhang

**Affiliations:** a State Key Laboratory of North China Crop Improvement and Regulation, Key Laboratory of Hebei Province for Plant Physiology and Molecular Pathology, College of Life Sciences, Hebei Agricultural University, Baoding, Hebei, China; b Pear Engineering and Technology Research Center of Hebei, College of Horticulture, Hebei Agricultural University, Baoding, Hebei, China; c Ministry of Education, Key Laboratory of Molecular and Cellular Biology, Hebei Collaboration Innovation Center for Cell Signaling, Hebei Key Laboratory of Molecular and Cellular Biology, College of Life Sciences, Hebei Normal University, Shijiazhuang, Hebei, China; d College of Life Sciences, Hengshui University, Hengshui, Hebei, China; e College of Plant Protection, Hebei Agricultural University, Baoding, Hebei, China; f College of Agronomy, Hebei Agricultural University, Baoding, Hebei, China

**Keywords:** Lateral root, auxin, receptor kinase, hormone, meristem

## Abstract

Lateral roots (LRs) are an important part of plant root systems. In dicots, for example, after plants adapted from aquatic to terrestrial environments, filamentous pseudorhizae evolved to allow nutrient absorption. A typical plant root system comprises a primary root, LRs, root hairs, and a root cap. Classical plant roots exhibit geotropism (the tendency to grow downward into the ground) and can synthesize plant hormones and other essential substances. Root vascular bundles and complex spatial structures enable plants to absorb water and nutrients to meet their nutrient quotas and grow. The primary root carries out most functions during early growth stages but is later overtaken by LRs, underscoring the importance of LR development water and mineral uptake and the soil fixation capacity of the root. LR development is modulated by endogenous plant hormones and external environmental factors, and its underlying mechanisms have been dissected in great detail in Arabidopsis, thanks to its simple root anatomy and the ease of obtaining mutants. This review comprehensively and systematically summarizes past research (largely in Arabidopsis) on LR basic structure, development stages, and molecular mechanisms regulated by different factors, as well as future prospects in LR research, to provide broad background knowledge for root researchers.

## Introduction

The root system is a general name for the plant root. The root that develops from the seed radicle is called the primary root. The primary root develops many branching roots, called secondary or lateral roots (LRs), at a specific angle with the primary root under the joint action of internal and external factors. LRs can further produce secondary LRs. Aboveground parts of plants, such as the hypocotyl, stem, or leaves, can also develop adventitious roots under appropriate conditions that integrate endogenous plant hormones and/or external stimulation. Many dicotyledonous plants, such as Arabidopsis (*Arabidopsis thaliana*), rapeseed (*Brassica napus*), tomato (*Solanum lycopersicum*), and carrot (*Daucus carota*), have an obvious primary root from which lateral roots develop upon stimulation by internal hormones, environmental factors, and nutrient supply and quota.

Plant roots not only serve as physical anchors in the soil but also support the uptake of water and micronutrients from the soil to sustain the growth and development of the aboveground plant tissues, including the reproductive organs. The LRs that develop from a primary root determine the structure and function of the plant root systems. The regulation of LR growth and development is complex, and there have been few systematic reviews in this area. This review is designed to fill this gap and help researchers better understand existing research on LRs.

### Basic structure of the plant root systems

Based on morphological characteristics, plant root systems can be divided into two types. Most dicotyledons have a primary root system, as illustrated in the model plant Arabidopsis, with a long, well-developed primary root and clear branching of LRs. In contrast, most monocotyledons have a fibrous root systems, as seen in rice (*Oryza sativa*). The main root of the fibrous monocot root stops growing or even dies early in development, but many adventitious roots and crown roots grow from the base of the hypocotyl and the lower stem nodes. The length and thickness of each root is similar, and they form the root system together with the seed root. In maize (*Zea mays*), post-embryonic root development starts with the formation of the primary root and seminal scutellar roots and then continues with the formation of adventitious crown roots, brace roots, and LRs.^1^ By contrast, the root system of a typical dicotyledonous seedling includes a primary root, LRs, and adventitious roots.^2,3^

### Formation of LRs

LRs are an essential part of the root systems. In dicots, LRs are clearly more numerous and explore more soil surface area than the primary roots, with their exact spatial range dictated by how much the LRs develop. In monocots, LRs constitute almost the entirety of the fibrous root system. Therefore, the physiological function of the entire root system is largely determined by the extent of LR development. Many plants form large aboveground parts to maximize sunlight capture and distribute carbon backbones via their vascular bundles, but they also need strong anchoring support from their roots, which is provided by LRs, as they account for most of the root system. The developmental plasticity of LRs may be used as a marker for a plant’s ability to adapt to changes in soil conditions. LR development occurs post-embryonically, but its internal cellular structure is identical to that of the primary root. In Arabidopsis, a typical dicot, the formation of a LR can be divided into eight stages.^4^

In stage I, the LR primordium begins to form. Near the root tip, columnar pericycle cells go through cell division perpendicular to the polar direction of Arabidopsis, resulting in 8 to 10 cells of the same size, which quickly increase radially. During the second stage, the central cells originating from stage I undergo a periclinal division to form an outer layer (OL) and an inner layer (IL), followed by another round of periclinal division of the outer cells during stage III to form two layers of outer layer cells (OL1 and OL2). In stage IV, the inner layer cells undergo a periclinal division to form two layers of inner layer cells (IL1 and IL2), and the LR primordium breaks through the endodermis of the primary root. During stage V, the middle cell of the outer layers (OL1 and OL2) divides three times, while the inner cells grow radially, pushing the outer cell outward and forcing the LR primordium to enter the primary root cortex. At stage VI, the outer cells divide; the inner cells continue to elongate along the root radius and take on the characteristics of vascular bundles; the epidermis, cortex, and endodermis form at the periphery of the LR primordium; and the LR primordium enters the primary root epidermis. During stage VII, the cell division and differentiation become more complex, with most divisions occurring perpendicularly to the primary root axis, allowing the LR primordium to grow outward through the epidermis. Finally, at stage VIII, each part of the LR primordium becomes established as an LR.^5^

### Formation of new meristems in LRs

Each LR primordium protruding from the epidermis of the primary root has its own root apical meristem (RAM), just like any primary root. The meristem contains a quiescent center (QC) with low mitotic activity and stem cells that surround the QC and can later differentiate into the various root structures.^6,7^ Many regulators also play a crucial role in primary root growth and development.

The LR primordium takes on characteristics of a meristem as early as when it is composed of three to five cell layers.^8^ During stage VI, three cell layers later give rise to the epidermis, cortex, and endodermis; the assumed stele tissue; and the potential root cap at the tip of the LR primordium. The establishment of each new meristem is closely related to the generation of an auxin maximum at the position of the future LR primordium.^9,10^ This process requires the relocation of the PIN-FORMED (PIN) family of auxin efflux carriers, mainly PIN1.^11,12^ PIN1 can be detected in root cells starting at the earliest stages of LR development. Indeed, from stage I to stage VIII, PIN1 accumulates in all except the outermost cells of the LR primordium. After the LR has matured, PIN1 localization returns to the typical pattern seen in the primary root, with accumulation in the vascular tissue.^13^ Many factors can regulate the phosphorylation state of PIN1, including the kinase PINOID (PID), Protein Phosphatase 2A family members (PP2As), Mitogen-Activated Protein Kinase 6 (MPK6), and MAPK Kinase 7 (MKK7), leading to changes in PIN1 polar location and thus affecting the development of lateral branches and roots.^14–16^ During LR growth, another type of hormone, cytokinin (CK), can induce the removal of PIN1 from the plasma membrane in a specific region by regulating PIN1 endocytosis, resulting in the depletion of PIN1 from the cell membrane.^12^ CK-regulated PIN1 polar localization is also related to the degree of PIN1 phosphorylation.^11^

The effects of three PLETHORA (PLT) transcription factors, PLT3, PLT5, and PLT7, during LR outgrowth are also crucial to LR development.^17^ In the *plt3plt5plt7* triple loss-of-function mutant, the morphology of the LR primordium, the auxin response gradient, and the expression of meristem/tissue recognition markers are impaired during the transition between phase I and phase II due to the circumferential cell division of “symmetry destruction,” in which the cells first obtain different identities on the paraxial and radial axes. In particular, during LR growth, *PLT1, PLT2*, and *PLT4*, genes that are usually expressed later than *PLT3, PLT5*, and *PLT7*, are not induced in the mutant primordium, resulting in a “PLT null” LR primordium. Reintroducing any PLT branch members into the *plt3plt5plt7* mutant primordium completely restores the layer identity of the second stage and repairs the mutational defects in meristem and tissue establishment. Therefore, any of the six *PLT* genes can activate the formative cell divisions that lead to de novo meristem establishment and tissue patterning associated with a new growth axis.^18^

### Regulatory mechanisms underlying LR development

In general, only a few pericycle cells can develop into LRs, suggesting the existence of regulatory mechanisms that determine which cells will form LRs.^19^ Plant hormones are involved in the regulation of LR development. In addition, external environmental factors such as water availability and nitrogen and phosphorus status also contribute to regulating LR formation to adapt to a changing environment.

### Regulation of LR development by auxin

While multiple plant hormones regulate LR development, the most important growth regulator is auxin.^20^ Auxin concentration changes rhythmically with a period of about 6 hours in the basal meristem, which is in agreement with the period of LR initiation.^21,22^ The polar auxin transport mutation *auxin resistant 1–7* (*aux1-7*) abolished this rhythm in auxin concentration and produced fewer LRs. When the auxin concentration of the basal meristem increases on one side, the columnar pericycle cells in the corresponding xylem pole begin to divide to form an LR primordium.^23^ In Arabidopsis, INDOLE-3-ACETIC ACID INDUCIBLE14 (IAA14) is the most important transcriptional regulator of auxin signaling during LR development. In plants carrying the *IAA14* gain-of-function allele *slr-1* (*solitary root1-1*), anticlinal divisions fail to occur in pericycle cells and LRs were completely underdeveloped.^24^ However, the loss-of-function mutant allele *iaa14-1* resulted in no obvious phenotypic changes, indicating that other IAA proteins may also participate in auxin-mediated LR development.^25–28^

IAA family members regulate auxin signaling by binding to or releasing AUXIN RESPONSE FACTORs (ARFs). In the context of LR development, IAA14 binds to *ARF7* and *ARF19* and blocks their activation of transcription. In agreement with this result, the *arf7arf19* double mutant exhibits a similar phenotype to the *slr-1* mutant, with a reduced number of LRs.^25–29^ However, in contrast to the complete loss of LRs in *slr-1*, the *arf7arf19* double mutant can form a few LRs, indicating that other ARF proteins also regulate LR development.^30^ In addition, LR development can be restored by overexpressing the genes encoding the transcription factors LATERAL ORGAN BOUNDARIES-DOMAIN16 (LBD16) and LBD29 in the *arf7arf19* double mutant. LBD family members are plant specific and play an important role in the development of lateral tissues. The *LBD16* and *LBD29* promoters contain binding sites for ARF7 and ARF19, making them direct targets of ARF7 and ARF19.^31^

SnRK1 (Snf1-RELATED-KINASE1) regulates LR formation by phosphorylating the bZIP63 (BASIC LEUCINE ZIPPER63) transcription factor, which can then directly bind and activate the promoter of *ARF19*; ARF19 is the key regulator of LR initiation.^32^ Arabidopsis seedlings that were cultured under different light and nutritional conditions to explore the effect of energy homeostasis on root structure, weak light, or short-term unexpected darkness (uD) showed increased emerged LR density (eLRD), but with little change in the length of the primary root.^32^ Measurement of the sucrose, glucose, and fructose contents and the expression of the energy stress–related gene *DIN6*/*ASN* (*DARK-INDUCED6*/*APARAGINE SYNTHETASE1*) in seedlings under different conditions confirmed that uD could instantaneously disrupt energy metabolism, and short-term uD could reduce soluble sugar and trehalose 6-phosphate contents, resulting in the expression of low-energy-stress genes. *DIN6*/*ASN* is a downstream gene of the central metabolic kinase SnRK1. To explore the effect of SnRK1 on LR formation, the authors created *snrk1α1* and *snrk1α2q* knockout mutants. The eLRD of the *snrk1α1* mutant decreased significantly after uD treatment, showing that *snrk1α1* acts to maintain LR activation after stress and SnRK1 is a required participant in the regulation of LR density during energy disturbance.^32^ Compared with those of the wild type, the primary root length and eLRD of the *bzip63* mutant increased, and LR formation induced by weak light also decreased. Therefore, bZIP63 is necessary for the increase in eLRD during short-term disturbance of energy steady state.^32^ The promoter of *ARF19* contains a G-box *cis* element (G-box1) and is a target of bZIP63 binding, as further confirmed by chromatin immunoprecipitation PCR (ChIP-PCR). The *arf19* mutant no longer induced eLRD in short-term darkness, indicating that ARF19 is also necessary for this response. These results indicate that SnRK1, bZIP63, and ARF19 together regulate LR development during energy homeostasis disturbance. This study thus provided mechanistic insights into how energy shapes the agronomically important root system.^32^

Auxin signaling mediated by various auxin/indole-3-acetic acid compounds (Aux/IAAs) and ARFs regulates LR development by controlling the expression of downstream genes.^33–35^ *LATERAL ROOT PRIMORDIUM1* (*LRP1*), a member of the *SHORT INTERNODES/STYLISH* (*SHI*/*STY*) family, has been identified as an auxin-inducible gene. *LRP1* is expressed in all stages of LR development except in the primary root, and its expression is regulated by histone deacetylation in an auxin-dependent manner. LRP1 acts downstream of the auxin response Aux/IAA ARF module during LR development. Auxin-mediated LRP1 induction is lost in the emerging LRs of *slr-1* and *arf7arf19* mutant roots. In plants treated with the auxin transport inhibitor *N*-1-naphthylphthalamic acid (NPA), LRP1 helps regulate the formation of LR after the normal and asymmetric division of LR founder cells.^36^ Overexpression of *LRP1* (*LRP1-OE*) resulted in an increase in the number of LR primordia in phases I, IV, and V, resulting in a decrease in LR density, indicating that *LRP1* is involved in LR primordium development. *YUCCA4* (*YUC4*), whose expression is induced by LRP1, is involved in auxin biosynthesis and contributes to the increase in endogenous auxin accumulation in *LRP1-OE* roots. LRP1 interacts with the SHI, STY1, SRS3, SRS6, and SRS7 proteins of the SHI/STY family, indicating that these proteins may play redundant roles in root development. Deacetylation of auxin and histone affects *LRP1* expression and plays a role in processes downstream of LR, creating an auxin response module that negatively regulates the development of LR primordia by regulating the dynamic balance of auxin in Arabidopsis.^37^

Auxin synthesized from the LR primordium promotes cell separation near the tip of the primordium to spatially accommodate its growth. As the LR develops from the pericycle cells in the inner layer of the primary root, it needs to break through multiple cell layers: three layers in Arabidopsis (endodermis, cortex, and epidermis), and more than three layers in other plants such as rice.^38^ Proteins related to cell wall remodeling play an important role in the breakthrough of LRs. The gene encoding the auxin influx carrier LIKE AUX1 3 (LAX3) is expressed specifically near the LR primordium to modulate the expression of several cell wall remodeling proteins, including PECTIN LYASE2 (PLA2), POLYGALACTURONASE (PG), EXPANSIN17 (EXP17), and GLYCOSYL HYDROLASE17 (GLH17).^39–41^ PLA2 and PG are involved in the degradation of demethylated pectin in the cell wall. EXP17 is another component of the plant cell wall that lacks enzymatic activity but can nevertheless relax the plant cell wall.

The normal elongation of LRs depends on normal polar auxin transport. Mutations in the gene encoding the auxin transporter MULTIDRUG RESISTANCE PROTEIN1 (MDR1) do not affect the initiation of LRs in Arabidopsis, but do prevent root elongation. Auxin concentrations are normal in the primary root tip of the *mdr1-1* mutant, but much lower in LR tips, compared to those in the wild type.^42^

Studies have shown that an H3K27 methyltransferase, CURLY LEAF (CLF), inhibits LR formation by depositing inhibitory H3K27me3 markers on the chromatin of the key polar auxin transporter gene *PIN1*.^43^ This found that the H3K27me3 demethylase REF6 promotes the initiation of LR primordia and the emergence of LRs. REF6 binds directly to the chromatin of *PIN1, PIN3*, and *PIN7*. Dysfunction of REF6 resulted in elevated H3K27me3 levels on *PIN1/3/7* and inhibited PIN gene expression. Genetic analysis of *clfref6* double mutants showed that there was antagonism between CLF and REF6 in their effects on LR formation. These results suggest that H3K27 methylation and demethylation activities may be coordinated to ensure appropriate LR organogenesis.^44^

Ultraviolet B (UV-B) light inhibited the LR growth of Arabidopsis in a UV RESISTANCE LOCUS8 (UVR8)-dependent manner.^45,46^ Monomeric UVR8 inhibits auxin response in a tissue-autonomous manner, thereby regulating LR growth. Auxin and UV-B radiation have antagonistic effects on auxin-regulated gene expression. UVR8 physically interacts with MYB73 and MYB77 (MYB domain proteins 73 and 77) in a UV-dependent manner. UVR8 inhibits LR development by regulating MYB73/MYB77. When activated by UV-B light, UVR8 localizes in the nucleus, inhibits the DNA-binding activity of MYB73/MYB77, and directly inhibits the transcription of its target auxin response gene. The UV-B-dependent interaction between UVR8 and MYB73/MYB77 is an important module for integrating light and auxin signals that enables them to coordinately regulate root development.^47^

TARANI, also called UBIQUITIN PROTEASE14 (TNI/UBP14), is necessary for LR primordium development. The UBP14 protein maintains normal auxin response through ubiquitin recycling. A *tni* mutant in Arabidopsis, in which poly-ubiquitin hydrolysis is reduced, shows pleiotropic phenotypic defects, including reduction in LR numbers, due to the stabilization of several AUX/IAAs and reduced auxin response. The smaller number of LRs observed in *tni* mutants may be due to defects in primordium initiation or its subsequent elongation. Here, we tested this by labeling the LR primordia with a *pCYCB1;1::CYCB1;1-GUS* reporter and calculating the number of LRs at different developmental stages. The decrease in the activity of TNI, a marker of LR primordium, leads to the delay of LR primordium initiation, thus shortening the LR of *tni* seedlings.^48^

In addition to the classical auxin signaling pathway, there are other important auxin signaling pathways involved in LR development. For example, MITOGENACTIVATED PROTEIN KINASE14 (MPK14) positively regulates the development of LR. The MPK14-deficient mutant *mpk14* has a LR development defect phenotype.^49^ MPK14 can interact with ETHYLENE RESPONSE FACTOR13 (ERF13). Moreover, auxin has been found to activate MPK14, which then phosphorylates ERF13, induces ERF13 degradation, and thereby relieves the inhibition of LR development by ERF13. *35S:ERF13-MYC* overexpression plants have a phenotype of significantly reduced LR breakthrough, which is caused by inhibition of the transformation of LR primordia from phase IV to phase V.^49^ A screen for genes regulated downstream of ERF13 showed that ERF13 can participate in LR development by inhibiting the expression of 3-ketoacyl-CoA synthase16 (KCS16) and then affecting the synthesis of very-long-chain fatty acids (VLCFAs). Furthermore, VLCFAs could affect the degradation of pectin in the cortical cell wall at the LR primordium, thus affecting the transformation of LR primordium from phase IV to phase V.^49^ In summary, this study clarified that auxin phosphorylates ERF13 by activating MPK14 kinase activity, and the phosphorylated ERF13 is degraded by the 26S proteasome to release the expression of the downstream gene *KCS16* and promote the synthesis of VLCFAs. VLCFAs further influence the degradation of pectin in the cortical cell wall at the LR primordium, thus affecting the transformation of LR primordium from phase IV to phase V and the subsequent development of LRs. This study expanded the molecular pathway of auxin regulation of LR development and revealed, for the first time, the details of a downstream molecular mechanism whereby mitogen-activated protein kinase (MAPK) signal regulates LR development.^49^ This further enriched our understanding of the molecular regulatory network of auxin regulation of LR development.

Recent studies have reported the molecular mechanism whereby nonclassical auxin signal regulates the cell division pattern during lateral root development through receptor-like protein kinase transmembrane kinases (TMKs).^50^ Double mutants with functional deletion of TMK1 and TMK4 have serious LR development defects and are insensitive to auxin treatment. At the cellular level, they show serious disorders of LR primordium cell division. TMK1/4 specifically interact with and phosphorylate two kinases, MKK4 and MKK5, in the MAPK signaling pathway.^50^ In this pathway, MKK4 and MKK5 are reported to act upstream of MPK3 and MKP6 to participate in the regulation of asymmetric cell division and stomatal development.^51^ The researchers observed that MKK4/5 and MPK3/6 were also involved in auxin-mediated LR development.^50^ Meanwhile, both MKK4/5- and MPK3/6-inducible double mutants showed disordered division of LR primordium cells.^50^ Previous studies showed that auxin can trigger the phosphorylation of MPK3/6, but the specific mechanism involved was not explained.^52^ This study showed that TMK1/4 is involved in auxin-mediated phosphorylation of MPK3/6 and thereby suggested that auxin may help control the division of LR primordium cells, and thus LR development, by regulating the phosphorylation level of MKK4/5-MPK3/6 through TMK1/4.^50^ This discovery of a TMK1/4-MKK4/5-MPK3/6-mediated nonclassical auxin-regulated LR development pathway further improved our understanding of the molecular regulatory network involved in auxin regulation of LR development.^50^

### Regulation of LR development by other plant hormones

In addition to auxin, other plant hormones also regulate LR development, with most participating in crosstalk with auxin signaling. CKs are negative regulators of LR development and are generally antagonistic to auxin during plant growth and development. Mutations in CK signaling components, such as Arabidopsis response regulators (ARRs) or Arabidopsis histidine kinases (AHKs), can lead to an increase in LR number in Arabidopsis.^53,54^ Higher CK catabolism *in vivo* will decrease CK contents, which also results in more LRs.^55^ CK inhibits the development of LRs in pericycle cells in the xylem pole, and the expression of genes related to CK biosynthesis in these cells inhibits LR development. By contrast, the expression of CK catabolism-related genes in xylem polar pericycle cells eliminates the repression of LRs by CK.^56^ Exogenous application of CK restricts the progression of LR development to stages IV and V, mainly by affecting auxin-induced *PIN* expression and, thus, polar auxin transport.^57^

Abscisic acid (ABA) mainly acts in the late stage of LR development. ABA INSENSITIVE3 (ABI3) is an important transcription factor in ABA signaling. The formation of LRs is less sensitive to exogenous auxin or auxin transport inhibitors in the *abi3* mutant,^58^ whereas mutants in *ENHANCED RESPONSE TO ABA1* (*ERA1*) encoding a farnesyl-transtransferase display more lateral roots. ABA is therefore necessary for auxin-mediated LR development. ABA is also involved in the effects of inorganic salts on LRs. High concentrations of nitrate (NO_3_^–^) can inhibit the development of LRs just after the LR primordium protrudes from the primary root. Indeed, the inhibitory effect of NO_3_^–^ is lower in the ABA biosynthesis mutants *aba deficient1* (*aba1), aba2*, and *aba3* and the ABA-insensitive mutants *abi4* and *abi5*.^59^

Brassinosteroids (BRs) and auxin play synergistic roles in the formation of LRs. The number of LRs is lower in the BR receptor mutant *br-insensitive1* (*bri1*), and exogenous BR application seldom changes auxin concentration at the root tip in wild-type seedlings. BRs appear to promote LR development by promoting polar auxin transport.^60^

There has been no in-depth study on the relationship between gibberellins (GAs) and LR development, although several reports have shown that GAs can affect LRs. GA-deficient pea (*Pisum sativum*) plants produce fewer nitrogen-fixing nodules and LRs than wild-type plants.^61^ Similarly, primary root growth is inhibited in Arabidopsis GA-deficient mutants, but their LR phenotypes were not reported in detail.^62^

Ethylene (ETH) negatively regulates the formation of LRs in Arabidopsis by affecting polar auxin transport.^63^ Exogenous application of ETH precursors decreases the number of LRs in the wild type, as does higher endogenous ETH biosynthesis in the *ethylene overproducer1* (*eto1*) mutant. By contrast, the ETH-insensitive mutants *ethylene triple response1* (*etr1*) and *ethylene insensitive2* (*ein2*) produced more LRs. It is worth noting that higher ETH levels enhance polar auxin transport, indicating that ETH has a positive regulatory effect on this process. Testing various mutants lacking individual auxin transporters identified AUX1 as the ETH target. Exogenous application of a high ETH concentration inhibits the formation of LR primordia but promotes the growth of existing LR primordia, while low ETH concentrations promote the initiation of LR primordia.^64^

Methyl jasmonate (MeJA) can also promote LR development. Treatment with exogenous MeJA can increase the expression levels of *ANTHRANILATE SYNTHASE ALPHA SUBUNIT1* (*ASA1*), which encodes a key rate-limiting step in auxin biosynthesis, leading to higher auxin contents *in vivo* that then promote LR genesis. MeJA can also affect local auxin accumulation in roots by inducing the expression of *PIN1, PIN2*, and *AUX1*.^65^ MeJA has also been shown to regulate auxin contents via the transcription factor ERF109 and its downstream target genes *ASA1* and *YUCCA2* (*YUC2*), which encode auxin biosynthetic enzymes.^66^

A more recently identified plant hormone, strigolactone (SL), has attracted attention by negatively regulating the number of lateral buds in aboveground tissues, but it can also regulate the development of LRs. SL mainly negatively regulates LR development, in line with the greater number of LRs in SL biosynthesis or signal transduction mutants. Conversely, the exogenous application of the SL analog GR24 diminishes the number of LRs that form in wild-type Arabidopsis seedlings.^67^

### Role of polypeptide hormones in LR development

Polypeptide hormones regulate the growth and development of plant LRs,^68^ including the CLE (CLAVATA3/EMBRYO SURROUNDING REGION (ESR)) family, the RGF (ROOT MERISTEM GROWTH FACTOR)/CLEL (CLE-like)/GOLVEN family, the CEP (C-Terminally Encoded Peptide) family, and the IDA (INFLORESCENCE DEFICIENT IN ABSCISSION) family. The polypeptide hormones CIF1 (CASPARIAN STRIP INTEGRITY FACTOR1) and CIF2 are necessary for the formation of intact Casparian strip, which helps control water and salt transport from the roots to the rest of the plant.^69^ *CIF2* is expressed at the beginning of LR primordia formation, and its encoding peptide restrains LR formation.^70^ CIF1 and CIF2 are often sensed by a receptor-like protein kinase (RLK) on the cell surface to initiate signal transduction, thus regulating plant growth and development.

Cell-to-cell and cell-to-environment communication play decisive roles during LR growth and development. RLKs are single-transmembrane-domain proteins located at the cell surface that perceive signals from the environment or neighboring cells.^71^ RLKs play a vital role in LR growth, development, and adaptation the environment. For example, Arabidopsis CRINKLY4 (ACR4) is homologous to the RLK CRINKLY4 (CR4) from maize. ACR4 regulates pericycle cell divisions during LR initiation.^72^ Two other RLKs, HAESA (HAE) and HAESA-LIKE2 (HSL2), regulate cell wall remodeling and degeneration when LRs break through the endodermis, cortex, and epidermis of the primary root.^73^ When plants experience nitrogen deficiency, CEPRECEPTOR1 (CEPR1) and CEPR2 can sense CEP polypeptide hormones to regulate the development of LRs.^74^ CLAVATA1 (CLV1) and PHLOEM INTERCALATED WITH XYLEM (PXY) also play an important role in regulating the development of LRs.^68,75,76^

Some CLE proteins are closely involved in LR development. Under nitrogen-deficient conditions, *CLE1, CLE3, CLE4*, and *CLE7* expression is induced in the primary root pericycle in Arabidopsis. The overexpression of *CLE* blocks the formation of LRs, indicating that CLE1, CLE3, CLE4, and CLE7 may inhibit LR formation. Under the same conditions, the *clv1* mutant exhibits longer LRs, indicating that CLV1 acts as a receptor for small CLE peptides. In fact, exogenous administration of small CLE3 peptides inhibits the formation of LRs in wild-type but not *clv1* seedlings, indicating that the inhibition of LR formation by CLE depends on CLV1.^75^ These results suggest that CLE peptides whose encoding genes are expressed in the pericycle are secreted extracellularly and may be sensed by CLV1 in adjacent phloem cells, regulating downstream signals to inhibit LR formation.^68,75^

CEP peptides, which were identified by bioinformatics and high-performance liquid chromatography (HPLC), affect the development of primary roots and LRs by blocking the division and growth of RAM cells.^77^ In the legume barrel clover (*Medicago truncatula*), MtCEP1 negatively regulates LR development.^78^ Arabidopsis *cep3* mutants form more LRs under various abiotic stresses, such as nitrogen deficiency, salt stress, and osmotic stress.^79^ Arabidopsis *CEP1* to *CEP5* are expressed mainly in LR primordia and less so in aboveground tissues.^80^ The overexpression of *CEP* genes also prevents the development of the primary root and LRs, as well as morphogenesis of aboveground tissues. Notably, the exogenous application of CEP1, CEP3, CEP5, and CEP9 disrupts the development of LRs.

Two receptor-like protein kinases, CEPR1 (also named XYLEM INTERMIXED WITH PHLOEM1 XIP1) and CEPR2, can bind to different CEPs.^74,81^ Consistent with the function of CEPs in inhibiting LR development, the *cepr1* single mutant and the *cepr1cepr2* double mutant produce elevated numbers of LRs.^74^

The LR protrudes from the endodermis, cortex, and epidermis cell layers of the primary root and must separate these cells before it can emerge into the neighboring soil.^5^ This protrusion is similar to the abscission of plant organs, with IDA, for instance, participating in both floral organ abscission and protrusion of LRs.^73,82^ The accumulation of auxin in the overlapping cortex and epidermal cell layers of LRs can induce the degradation of SLR/IAA14, allowing the newly released ARFs to promote *IDA* expression.^73^ IDA is perceived by HAE/HSL2, which causes cell wall relaxation by inducing the expression of cell wall remodeling enzymes.^73,83^ In agreement with the above signal transduction pathway, cell wall pectin of adjacent cells cannot be degraded in the *ida* single mutant or the *haehsl2* double mutant, preventing the separation of adjacent cells and thus blocking the protrusion of LR primordia through the cortex and epidermis.^73^

Rapid alkalinization factors (RALFs) affect cell growth by regulating calcium (Ca^2+^) responses, MAPK signaling, and alkalinization, and also participate in LR development. The overexpression of *RALF1* lowers the density of LRs and partially represses *RALF1* expression to promote the protrusion of LRs, indicating that RALF1 negatively regulates LR protrusion.^84^ RALF1 and other RALFs, such as RALF19 and RALF34, also participate in the initiation of LRs,^84,85^ as does RALF-LIKE34 (RALFL34). Indeed, *RALFL34* is expressed in the pericycle cells of the protoxylem. The *ralfl34* mutant shows an ‘aberrant’ cell division pattern and a higher LR density, indicating that RALFL34 plays a role in pericycle cell division during LR initiation.^86^

THESEUS1 (THE1) is the RALF34 receptor, and together they fine-tune the initiation of LRs. The *ralf34* and *the1* mutants exhibit an increase in the proportion of LRs at stage I and abnormal asymmetric divisions of established cells.^86,87^ The THE1-RALF34 signaling module affects the initiation of LRs by regulating the integrity of cell walls, which depends in part on the RLK FERONIA (FER). The *fer-4* mutant displays an abnormal columella stem cell morphology and produces more LRs, and its roots have a slow gravity response, phenotypes that are reminiscent of auxin polar transport defects. The polarity of PIN2 is altered in the *fer-4* mutant, which is accompanied by a shortening of actin filaments, indicating that FER regulates actin-mediated PIN2 polar localization, which is then responsible for the defects in LR development and gravity response.^68,88^

TARGET OF LBD SIXTEEN2 (TOLS2) is a small secreted peptide consisting of 11 amino acids in its processed, mature form. Its encoding gene is expressed in LR precursor cells and is induced by auxin. This direct target of LBD16 can inhibit LR initiation.^89^ PUCHI is an APETALA2 (AP2)-type transcription factor that can regulate LR development; *PUCHI* expression is induced by TOLS2 and requires RLK7, indicating that *PUCHI* is a downstream target of RLK7 signaling.^90–92^ The TOLS-RLK7-PUCHI signaling module selects the cells within the root tip oscillating zone and inhibits the initiation of LRs, thus ensuring a proper spatial distribution of LRs along the primary root.^68,92^

The Arabidopsis genome encodes 11 CLEL family members. CLEL is also called RGF and GOLVEN (GLV), reflecting the multiple roles played by this family.^93,94^ Ten of the 11 members of this gene family are expressed during LR development, and the overexpression of most members leads to LR defects. For example, overexpression of *CLEL2, CLEL6*, and *CLEL7* delays the development of LRs by interfering with the division of pericyclic cells and inhibiting LR initiation.^68,95,96^ Treatment of wild-type seedlings with exogenous RGF1 (also called CLEL8) can reduce the number of LRs. Importantly, perception of the RGF1/CLEL8 signal requires receptors from the RGF1 INSENSITIVE1 (RGI1) family, as the *rgi1rgi2rgi3rgi4* quadruple mutant does not respond to RGF1 treatment.^97^

Phytosulfokine (PSK) is a five-amino-acid sulfated peptide that affects the dedifferentiation and proliferation of plant cells. There are five *PSK* genes in Arabidopsis, all of which are expressed in LR primordia.^98^ The regulation of primary root length by PSKs is mainly perceived through PHYTOSULFOKIN RECEPTOR1 (PSKR1) and PKSR2, while exogenous PSK treatment can affect the protrusion and total length of LRs by increasing cell number, but this process is independent of PSKR1, indicating that PSK may bind to other receptors to regulate the development of LRs.^68,98^

### Regulation of LR development by nutrient status

The plant root system integrates internal developmental processes and biotic and abiotic factors. The plasticity of the root system is an important feature shaping its growth and development. For instance, a localized supply of nitrogen in the form of nitrate can promote LR development. However, the initiation and development of LRs are repressed in high-nitrogen ^99,100^ and high-carbon/high-nitrogen (C/N) environments.^101^ Regulation of root development by nitrogen status appears to play different roles at different stages of LR development. Indeed, the inhibition of LR development caused by a high C/N ratio likely takes place during LR initiation, as a high C/N ratio limits auxin transport.^101^ By contrast, the inhibition of LR elongation induced by high N occurs at later stages when LRs elongate, after the LR meristem has formed.^59^ While the number of LR primordia formed under different N concentrations is similar, the resulting LR elongation varies, with shorter LRs under high N. Local N supply only raises the LR elongation rate but has little effect on LR number.^99^ Studies in Arabidopsis showed that the promotion of LR formation by local N supply depends on the perception of the NO_3_^–^ signal by the LR, which leads to an increase in the activity of the LR meristem.^100,102^ The MADS box transcription factor ARABIDOPSIS NITRATE REGULATED1 (ANR1) is an important regulator of NO_3_^–^ signaling.^102^ NITRATE TRANSPORTER1.1 (NRT1.1, also named CHLORINA1 CHL1) is both a NO_3_^–^ transporter and receptor (a transceptor).^103^ NRT1.1 regulates the growth of new primary roots and LRs,^104^ the inhibition of *NRT2.1* expression by NO_3_^–^,^105^ and the promotion of seed germination by NO_3_^–^.^106^ LR initiation is also affected when seedlings are grown on low-phosphorus soil, although already established LR primordia appear to grow better; like very high N, low phosphorus supply inhibits LR initiation.^107^ LRs in barley (*Hordeum vulgare*) are longer in soils rich in potassium, whereas potassium deficiency blocks LR development in Arabidopsis.^108^ In nitrogen-poor areas of the soil, the auxin transport function of NRT1.1 is activated, resulting in inhibition of root growth.^109,110^ Activated NRT1.1 induces the expression of the auxin receptor gene *AUXIN SIGNALING F-BOX3* (*AFB3*), thereby regulating primary root growth and LR density.^111,112^ Low nitrogen concentrations inhibit the growth of LR primordia by inducing the production of small CLE peptides.^75^ In addition, under low-nitrogen conditions, AGAMOUS-LIKE2 1 (AGL1) upregulates auxin biosynthesis genes, leading to increased auxin levels in the LR primordium and LR and thus stimulating LR primordium initiation and LR elongation.^113^

By contrast, mild nitrate deficiency positively affects LR elongation. However, both nitrate and ammonium salts (for the latter, via processes dependent on the ammonium transporter AMT1;3;,^114^ and especially their local levels, positively affect LR formation, and nitrogen deficiency usually reduces LR formation. This type of complex regulation can greatly reduce the cost of plant root metabolism, allowing the plant to devote more energy to root elongation when nitrogen is scarce but more energy to root initiation when nitrogen is more plentiful. In addition to nitrogen and phosphorus, other nutrients also affect root development, but little is known about the underlying regulatory mechanisms. Deficiency in sulfur, magnesium, or iron reduces LR density in Arabidopsis, while that of potassium, calcium, zinc, manganese, or boron increases LR density.^115,116^ Excessive manganese represses auxin biosynthesis and the expression of the auxin transporter genes *PIN4* and *PIN7*.^117^ Similarly, excessive iron inhibits the expression of *PIN2* and blocks LR formation,^118^ and it also induces *AUXIN RESISTANT1* (*AUX1*) expression and triggers root elongation.^119^

Nitrate can influence many aspects of plant growth and development, for example, promoting root growth and inhibiting the synthesis of secondary metabolites. A nitrate-induced NAC family ^120^ transcription factor, NAC056, promotes nitrate assimilation and root growth in Arabidopsis. NAC056 is a nuclear-localized transcription activator that is mainly expressed in roots and hypocotyls. A *nac056* mutant showed deficient root growth, whereas *NAC056* overexpression promotes LR initiation and nitrate deficiency tolerance. It was found that NAC056 regulates the expression of genes required for NO_3_^–^ assimilation and directly targets the key nitrate assimilation gene *NIA1*. In addition, mutation of *NIA1* inhibited LR development and nitrate tolerance of *35S:NAC056* transgenic plants. Therefore, NAC056 mediates the response of plants to environmental nitrate signals and promotes root growth in Arabidopsis.^121^

As water is essential for plant growth and development, plant roots have evolved different strategies to cope with drought. The uneven distribution of water in the soil also affects the formation of LRs. Indeed, plant roots can perceive extremely small changes in water availability in their surrounding environment and respond with changes in root configuration. Only the side of the primary root in contact with water produces a new LR, a process known as hydropatterning, which in Arabidopsis depends on the SUMO modification of the transcription factor ARF7. The modified ARF7 combines with IAA3 to form a protein complex that inhibits transcription, thus affecting the initiation of LR by negatively regulating the expression of auxin response genes.^122^ By contrast, if the soil lacks water, the formation of LRs is prevented, a process called xerobranching that is mediated by the ABA signaling pathway. However, hydropatterning is not affected in ABA signaling mutants. This model does not require the coordinated regulation of CK signal transduction and auxin biosynthesis, or the participation of ABA in arsenate-induced root growth inhibition.^123,124^ Plants adapt to different water levels in the soil by switching back and forth between hydropatterning and xerobranching, allowing their roots to make more effective use of water resources. In addition to being more tolerant to nitrogen starvation, maize lines with fewer but longer LRs are more tolerant to drought, indicating that the reduction of LR growth might help plants deal with general drought stress in a slightly arid environment.^125^

The uptake of water and inorganic salts depends to a large extent on the development of LRs. Rice is a typical silicophilic crop with a high silicon content. Silicon can enhance the toughness of leaves and significantly improves the resistance of rice. The absorption rate of silicon is much lower in the rice non-lateral-root mutant RM109 and is accompanied by a lower deposition of silicon in the leaf epidermis.^126^ Like those of other organs, the growth and development of LRs require building blocks and energy. In some cases, reducing the number of LRs is beneficial for nutrient uptake: For example, under drought conditions, maize plants with fewer LRs grow longer roots to explore deeper into the soil for a water source, whereas plants with many LRs are more limited to the moisture available at the soil surface.^125^

### Regulation of LR development by environmental factors

Once rooted in the soil, plants face a variety of environmental factors, including light and temperature, some of which influence root structure and LR growth. Arabidopsis shoots that perceive a drop in the red/far-red light ratio respond by repressing the formation of LRs. The abundance of the transcription factor ELONGATED HYPOCOTYL5 (HY5) increases in the LR primordium when the relative fluence of far-red light rises and requires the red/far-red photoreceptors phytochromes. Later studies demonstrated that HY5 affects LR formation by diminishing the abundance of the auxin transporters PIN3 and LAX3.^127^ Likewise, during rice root development, unilateral light exposure induces the formation of LRs on the illuminated side.

Plants typically experience salt stress concurrently with drought stress and osmotic stress. In Arabidopsis, salt stress results in root enlargement and shortening, while the number of LR primordia decreases, and the expression of some genes related to the cell cycle is repressed.^128^ The overexpression of chickpea (*Cicer arietinum*) *CAP2* (*Chickpea AP2*) in tobacco (*Nicotiana tabacum*) increases the number of LRs and enhances tolerance to salt stress and osmotic stress. The expression of many abiotic stress–related genes and auxin response genes related to LR development is also upregulated.^129^ Salt stress can induce the expression of *NAC2*, encoding a transcription factor involved in auxin and ETH signaling. *NAC2* overexpression changes the number of LRs in Arabidopsis, indicating that *NAC2* is an important factor mediating the inhibition of LRs by salt stress.^130^The Salt Overly-Sensitive (SOS) signaling pathway also plays an important role in plant resistance to salt stress. Under low-salt conditions, the sensitivity of LR development of the mutant *sos3-1* to salt stress increases, as a result of lower auxin levels at the primordia of cotyledons and LRs.^131^ These results illustrate the close relationship between plant salt stress responses and auxin-mediated LR development.

Drought also affects the development of LRs. When water availability in the soil is uneven, roots attempt to avoid dry areas and grow toward moisture.^132^ The addition of mannitol to the growth medium to simulate drought stress either induces the development of LRs in Arabidopsis or delays their development. Drought stress induces the accumulation of ABA and inhibits the initiation of LRs, which is consistent with the inhibition of LR formation by exogenous ABA. This process relies on the osmotic stress response protein LATERAL ROOT DEVELOPMENT2 (LRD2); LRD2 and ABA signaling further interact with auxin, in turn regulating LR initiation.^133^ To delve deeper into the complex mechanisms behind drought regulation of LR development, several Arabidopsis mutants with defects in drought-mediated repression of LR growth have been isolated. In the *dig3* (*drought inhibition of lateral root growth3*) mutant, drought or ABA has little effect on LR development compared to the wild type, indicating that DIG3 is necessary for ABA to inhibit LR growth.^134^

Sugars not only are the energy source and intermediate metabolites for plant growth and development, but also play a role in signal transduction by regulating the development of a variety of organs, including LR development. The mode of regulation of plant LR growth and development by sugars is currently thought to be mediated by the HEXOKINASE1 (HXK1) and glucose-TOR (TARGET OF RAPAMYCIN) signaling pathways. When glucose is not present in the medium, LR growth of both the *glucose insensitive2* (*gin2*) mutant and wild-type Arabidopsis is inhibited. Upon glucose addition, LR growth returns to normal in both genotypes, indicating that the activation of the RAM by glucose is not dependent on the HXK1 pathway.^135^ However, when Arabidopsis seedlings are subjected to different concentrations of glucose, the changes in the LRs and primary root of the *gin2* mutant are lower than those seen in the wild type, indicating that glucose imposes an effect on LR and primary root growth through a concentration gradient that relies on the HXK1 signaling pathway.^136^ In addition, glucose-TOR signaling contributes to the regulation of a series of growth processes, including LR development in plants.^137^ Rapamycin, an inhibitor of the TOR kinase, represses the signal emanating from glucose-TOR signaling. Wild-type Arabidopsis seedlings treated with rapamycin show an induction of LR growth that is largely comparable to that of estradiol-inducible *TOR* RNA interference lines.

The MEDIATOR (MED) complex plays a variety of functions in plant development, hormone signal transduction, and biological and abiotic stress tolerance through transcriptional coordination. MED12 and MED13 are involved in root structure formation and auxin and sugar reactions. *med12* and *med13* single mutants showed shoot and root phenotypes consistent with effects on auxin homeostasis, including changes in primary root growth, LR development, and root hair elongation.^138^ MED12 and MED13 are necessary to activate primary root cell division and elongation, auxin response, and stem cell niche (SCN) gene expression. It should be noted that most mutant phenotypes can be rescued by providing sucrose to the growth medium. The growth response of primary roots of wild-type and *med12, aux1-7*, and *med12aux1* single and double mutant plants to sucrose and to the auxin transport inhibitor NPA revealed a correlation between *med12* phenotype and the activity of auxin uptake permeability enzymes and showed that MED12 acted upstream of AUX1 in the growth response of roots to sugar.^138^

The spacing of plant LRs along the main root is driven by oscillatory signals, usually called the “root clock”,^139^ representing a pre-patterning mechanism that can be affected by environmental signals. Light is an important environmental factor that was previously reported to regulate the root clock. Light has been found to activate the transcription of the light morphology gene *HY1* to maintain high frequency and amplitude of the oscillation signal, resulting in the repeated formation of pre-branching sites. HY1 produced locally by peripolar cells in stem or xylem is sufficient to regulate LR branching. In addition, HY1 can induce the expression of *HY5* and its homolog *HYH* and regulates the intracellular localization and expression of auxin transporter as a signal body, so as to promote the accumulation of auxin in the oscillation region and stimulate LR branching. These basic mechanistic insights improved the understanding of the molecular basis of light-controlled LR formation and provide a genetic link between stem- and root-derived signals in the regulation of periodic LR branching.^140^

### Roles of gravity and mechanical signals in LR development

The spatial occurrence of LRs is also affected by gravity and mechanical factors.^23,141–143^ For example, a LR primordium^143^ can form on the outside of the gravity bend of the primary root. The auxin reporter construct *DR5:VENUS-YFP* (driving *Yellow fluorescent protein* (*YFP*) expression from the synthetic promoter *DR5*) showed auxin accumulation in pericycle cells at this location.^141,142^ However, it is difficult to explain how pericycle cells deviating from gravity can accumulate high concentrations of the hormone, as auxin transport typically follows the direction of gravity.^144,145^The high auxin accumulation in pericycle cells deviating from the gravity vector is thought to be due to the effect of mechanical factors on cell size. As a possible molecular mechanism, the gene encoding the auxin influx transporter AUX1 is highly expressed in pericycle cells, leading to the observed high accumulation of auxin.^142^It has also been reported that mechanical induction leads to the expression of auxin-responsive genes at the beginning of LR initiation, which requires the relocalization of PIN1 in the xylem cells adjacent to the columnar pericycle cells.^141^ Another pathway independent of auxin has also been proposed.^9,10^ The Arabidopsis *axr4-2* mutant has fewer LRs than the wild type, and the plants are generally smaller. In tensile tests, *axr4-2* has lower average tensile strength than the wild type, but between single plants, the single root of *axr4-2* with the strongest tensile strength per plant was stronger than that of the wild type.^146^

### Other regulatory factors affecting LR development

Other regulators modulate LR development. An Arabidopsis double mutant lacking *ARABIDILLO-1* and *ARABIDILLO-2* function forms fewer LRs than the wild type, while overexpression of either gene results in more LRs. Both proteins contain an F-box domain, which may regulate LR development by mediating the degradation of an unknown protein.^147^

The NADPH oxidase subunits RESPIRATORY BURST OXIDASE HOMOLOG D (RBOHD) and RBOHF negatively regulate LR development by acting on the pool of superoxide in the cell. In an Arabidopsis *rbohDrbohF* double mutant, superoxide radicals accumulate in the root, leading to higher peroxidase activity; the *rbohDrbohF* double mutant also produces more LRs.^148^

POLY(ADP-RIBOSE) POLYMERASEs (PARPs) are DNA repair enzymes that cope with DNA damage. The *parp1parp2* double mutant produces more LRs that elongate faster than in the wild type, which is accompanied by an upregulation of the expression of genes related to cell division; the mutant also displays a larger meristem.^149^

## Conclusion and prospects

**Figure 1.**
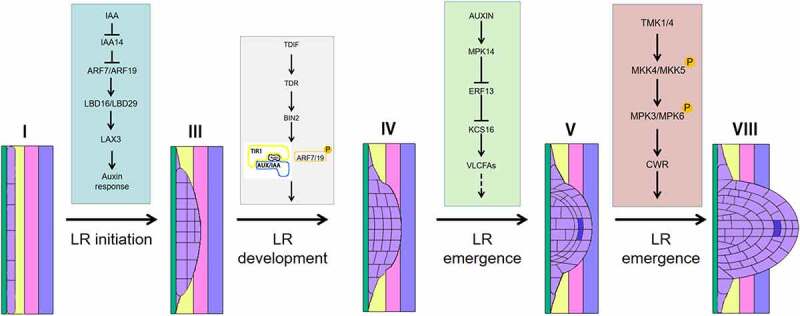
LR regulatory network in each developmental stage.

**Figure 2.**
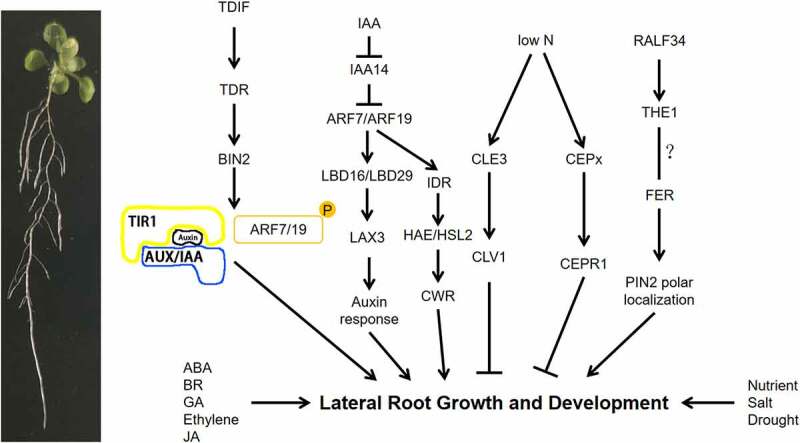
LR network regulation model.

The genes involved in LR development have been studied in detail in the model plant Arabidopsis (Figure 1). LRs develop post-embryonically but take on the same basic structure as primary roots. In Arabidopsis, LR development goes through four steps: initiation, primordium development and formation, activation of the lateral root apical meristem, and LR orientation and elongation.^150^ LR primordia originate from pericycle cells ^151^ outside the xylem at a certain distance from the primary root meristem. These cells from the pericycle, also known as primordial cells, undergo multiple rounds of cell division to form the incipient LR primordium. The primordial cells are not in the RAM, but rather in the differentiation zone. The cells at these sites have thus exited the mitotic cycle, but under proper stimulation they reenter the cell division cycle to carry out periclinal divisions, form LR primordia, and then break through the epidermis to form LRs.^152^ Once the LR primordium is formed, depending on the LR primordium meristem, it can develop into a mature LR. Therefore, the initiation of LRs is very critical (Figure 2).

Regulatory network of LR growth and development. Solid lines indicate positive (pointed arrows) or negative (flat-headed arrows) regulation.

Overview of the LR growth and development. A schematic of the auxin and receptor protein kinases regulatory network in the LR. Solid lines indicate positive (pointed arrows) or negative (flat-headed arrows) regulation. Question mark indicates there is no particularly clear regulatory relationship between genes.

The formation of LRs is a complex developmental process that is tightly regulated to facilitate nutrient and water uptake from the soil. For most plants, LR formation takes place after embryo development. The Arabidopsis middle root originates from pericycle cells ^36^ in the root elongation region near the pole of the protoxylem. After regaining totipotency, these pericycle cells undergo a series of divisions to form LR primordia. Each primordium then protrudes through the primary root epidermis and becomes a mitotic meristem from which the new LR will develop. The mechanisms behind the location (longitudinal positioning) and spatial distribution of LR primordia are not clear.^153^ Cell division was first observed at about 1.4 mm from the tip of the primary root for the first emerging LR.^19^ This length of growth corresponds to a time frame of 14 hours from when the central column pericycle cells of the protoxylem poles left the RAM.^19^ The LR provides a physical anchor for the plant and enables water and nutrient absorption, such that the development of more LRs increases the uptake capacity and surface area of the root system. Roots undergo a series of phenotypic changes under stress, including inhibition of primary root growth, development of more lateral and adventitious roots, and development of more root hairs.^154^ The above phenotypic changes are the result of plant adaptation to stress, for which LR initiation and development offers a good model system. Therefore, the study of the genes and regulation involved in LR development has important theoretical and practical significance for agricultural production.^155^

LRs are also of great significance to the normal growth of plants. In terms of regulatory mechanisms, a variety of regulatory factors, including various hormones, have been shown to participate. Despite these advances, several challenges remain, such as developing a comprehensive and accurate understanding of the regulatory network of LR development, which has yet to be constructed. In addition, to realize the full yield potential of crops, it will be necessary to further explain the interactions between LR development and the changing environment. To solve these problems, researchers need to further analyze and study the regulatory factors and mechanisms of LR development from a variety of research perspectives and by various technical means.

With the progress of technology, live-cell imaging ^156^ now enables us to track multiple molecular markers at the same time over long periods and observe the dynamic changes of molecules. Computational modeling ^157^ in biology can also help us simulate and predict the interaction between hormones that regulate LRs. RNA single-cell sequencing ^158,159^ and gene editing technology ^160^ can enable us to better decipher the mechanism of hormones regulating LR development.
